# *Cancer Borealis* Stomatogastric Nervous System Dissection

**DOI:** 10.3791/1207

**Published:** 2009-03-23

**Authors:** Gabrielle J. Gutierrez, Rachel G. Grashow

**Affiliations:** Volen Center for Complex Systems, Brandeis University

## Abstract

The stomatogastric ganglion (STG) is an excellent model for studying cellular and network interactions because it contains a relatively small number of cells (approximately 25 in *C. borealis*) which are well characterized. The cells in the STG exhibit a broad range of outputs and are responsible for the motor actions of the stomach. The stomach contains the gastric mill which breaks down food with three internal teeth, and the pylorus which filters the food before it reaches the midgut. The STG produces two rhythmic outputs to control the gastric mill and pylorus known as central pattern generators (CPGs). Each cell in the STG can participate in one or both of these rhythms. These CPGs allow for the study of neuromodulation, homeostasis, cellular and network variability, network development, and network recovery.

The dissection of the stomatogastric nervous system (STNS) from the Jonah crab (*Cancer borealis*) is done in two parts; the gross and fine dissection. In the gross dissection the entire stomach is dissected from the crab. During the fine dissection the STNS is extracted from the stomach using a dissection microscope and micro-dissection tools (see figure 1). The STNS includes the STG, the oesophageal ganglion (OG), and the commissural ganglia (CoG) as well as the nerves that innervate the stomach muscles. Here, we show how to perform a complete dissection of the STNS in preparation for an electrophysiology experiment where the cells in the STG would be recorded from intracellularly and the peripheral nerves would be used for extracellular recordings. The proper technique for finding the desired nerves is shown as well as our technique of desheathing the ganglion to reveal the somata and neuropil.

**Figure:**
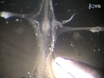


## Protocol

### 1. Gross Dissection

Place a crab on ice for 30 minutes to dull the sensory and motor systems of the animal. Surround it on all sides with ice. Gloves should be worn when handling the crab.
Prepare for the gross dissection by laying out the dissection pan and the tools you will use. Rongeurs, bone-cutting scissors, small scissors, toothed forceps, a spatula with a tapered edge, a black Sylgard-coated dish, and insect pins are needed.
Remove the crab from the ice bucket by its hind leg to prevent it from pinching and place it in the dissection pan.
Remove the claws first by twisting each one inward. Then remove each leg using the same twisting motion.
Using the rongeurs, remove the maxilla covering the mouth. Also remove the soft maxillule.
Using the rongeurs again, start from the lateral posterior end of the carapace and get a good grip on the edge of it. Clamp down with the rongeurs and break that piece of carapace off. Make sure to remove enough of the carapace to allow the spatula to enter the opening. Continue removing the edge of the carapace up to the eyes and repeat on the other side.
With the tapered side of the spatula, gently scrape the soft inner tissue away from the roof of the dorsal carapace. Keep the spatula in contact with the carapace to avoid damaging the STNS. Use the rongeurs to break away the parts of the carapace that have been separated from the tissue. Also separate some of the soft tissue from the ventral carapace making sure to separate any adductor muscles attaching the tissue to the carapace.
Break away the dorsal carapace as far posterior as the heart where there are two small ossicles protruding from the carapace. Cut as far anterior as possible. Stabilize the cephalon (the “face”) with one finger while breaking away the most anterior parts of the dorsal carapace.
On the ventral side of the carapace, pull out the mandibles one at a time by anchoring the rongeurs onto a mandible, twisting them outwards to dislodge the mandible from the attached epistome, and pulling it away. Two long ossicles with muscles should be attached to the extracted mandible if done properly.
Separate away the tissue from the sides of the cephalon near the eyes using the spatula. With small dissection scissors, cut across the tissue as near to the ridge of ossicle as possible layer by layer.
Rest the crab against a wall of the dissection pan and prop it up securing it in place (the claws may be used for this). Cut the labrum (“upper lip”) away from the epistome making sure not to leave any tissue attached. Using bone-cutting scissors make two diagonal cuts into the ventral carapace to remove the cephalon.
Take the labrum with toothed forceps and cut the labium (“lower lip”) away from the ossicle on the ventral side. Then gently lift the lip away from the remaining ventral carapace. Use the small scissors to separate the ventral part of the stomach from the tissue lining the carapace. When the pyloric ampullae are visible, cut the stomach away from the tissue on either side of it until the stomach is extracted from the crab.
Still holding the stomach by the lip with forceps, rest the stomach against the palm of the hand and pour saline into the mouth. This expands the stomach and makes it easier to cut open. Cut from the opening at the mouth down between the pylorus right through the ossicle between the ampullae. Then cut diagonally down through the two cardial branches. Cut off the tips of the three teeth inside of the stomach.
Place the stomach onto a large, black, Sylgard-coated dish with the inside of the stomach down. Pin down the five corners of the preparation with insect pins and fill the dish with cold saline, immersing the entire preparation (see figure 1).

### 2. Fine Dissection

, Micro-dissection tools are needed for the fine dissection. In particular, forceps and spring scissors are needed as well as a pin holder with a tungsten needle. A clear, Sylgard-coated, petri dish and some fine wire pins should also have been prepared.
Beginning on the lowest magnification, start by pinning down the flaps near the lip that used to be the mouth opening. Rearrange the rest of the pins to make the preparation taut and pin any other areas necessary.
Carefully lift the spotted hypodermis covering the lower part of the preparation and trim it across leaving a patch of hypodermis with two white dots.
Peel up the yellow, wispy, membranous tissue near the bottom of the preparation. The *mvns* are weakly attached to the underside of this tissue. Carefully separate the *mvns* from the yellow tissue as it is pulled up. Follow the yellow tissue up and cut alongside the brain.
Follow the thick processes exiting the brain that extend laterally. These are the commisural nerves. Once the end of the commissural nerve is reached, remove the mass of spongy, off-white tissue that has been cut away from the side of the brain.
Where the *ions *and* sons* meet the CoGs, cut away the muscle and tissue to reveal the length of the* ions *and* sons*.
Return to the spotted hypodermis patch and cut it off. This should leave an opening in the artery surrounding the STG. Cut through the opening to separate the two muscles that flank the STG. Lift the ends of these muscles and cut right underneath them to separate them from the artery and to expose the *alns *and* agns*. Separate the flanking muscles as far up as the cartilage lying over the *stn*.
Cut away the arterial tissue surrounding the STG down to where the *mvns*,* dgn*,**and* dvn* meet. Work down the dvn to the lvns and cut through the whitish, delicate tissue covering the *lvns*.
Locate the *psn* over the ossicle near the bottom of the preparation. The psn can be used to locate the *lvn* and *dlvn* junction.
Next follow the *dlvn* down to locate the* pyn *and* pdn*. The pyn often wraps over the pyloric ampulla to the opening. The *pdn* forks off of the *dlvn* and is between the pyloric ampulla and the cardio pyloric valve muscles. Leave some muscle attached to one of these nerves because they can be easily confused when the preparation is transferred to the clear Sylgard dish.
Once all of the nerves have been uncovered, sever any remaining connections between the STNS and the tissue and unwanted nerves of the stomach. Carefully move the STNS away from the rest of the stomach, cutting any missed connections.
Condition the clear Sylgard dish with the remaining mass of stomach tissue by rubbing it over the surface of the Sylgard until the Sylgard no longer feels too sticky or dry. Sylgard is hydrophobic and the STNS will adhere strongly to it if the Sylgard-coated dish is not conditioned in this manner.
Add some cold saline to the dish. Grab the upper ends of the commissural nerves with forceps and bring the STNS to the clear Sylgard dish.
Pin the STNS down onto the Sylgard. Sever the brain from the commissural nerves and use the minuten pins to secure the four ends of the CoGs down first. Ensure that the preparation is right side up by checking that the *ivn* is pointing up away from the Sylgard. The *dgn* should exit slightly beneath the STG when the STG is right-side up. Pin the rest of the nerve ends down with the fine wire pins.
Clean away any remaining muscle or tissue from the STNS.
Desheath the STG using a pin holder and a slightly hooked, fine, tungsten needle. Make a small hole in a corner of the STG sheath away from the cell bodies and use that hole to get between the layers of sheath. Carefully cut a flap of separated sheath to expose the STG neuropil and cell bodies.
Pin any flaps of sheath down to increase accessibility to the cell bodies and to stabilize the STG for intracellular recordings. Re-pin the rest of the STNS ensuring that each nerve is taut and well spaced for extracellular recording (see figure 3).

### 3. Results

Ideally, all of the nerves should be free of nicks and damage, particularly the ones that will be recorded from. None of the nerves should be tangled or twisted. The STG should be intact with all of the cells arranged in a beard formation around the neuropil. The intact STNS is bilaterally symmetrical and looks like a homunculus with the lvns as the *legs*, the *mvns* as the arms and the anterior end as the head.

### Abbreviations:

**Table:** 

STNS	Stomatogastric nervous system
STG	Stomatogastric ganglion
CoG	Commissural ganglion
OG	Oesophageal ganglion
mvn	Median ventricular nerve
ion	Inferior oesophageal nerve
son	Superior oesophageal nerve
agn	Anterior gastric nerve
aln	Anterior lateral nerve
dgn	Dorsal gastric nerve
dvn	Dorsal ventricular nerve
lvn	Lateral ventricular nerve
psn	Posterior stomach nerve
pyn	Pyloric nerve
pdn	Pyloric dilator nerve
dlvn	Dorsal branch of the lateral ventricular nerve
ivn	Inferior ventricular nerve


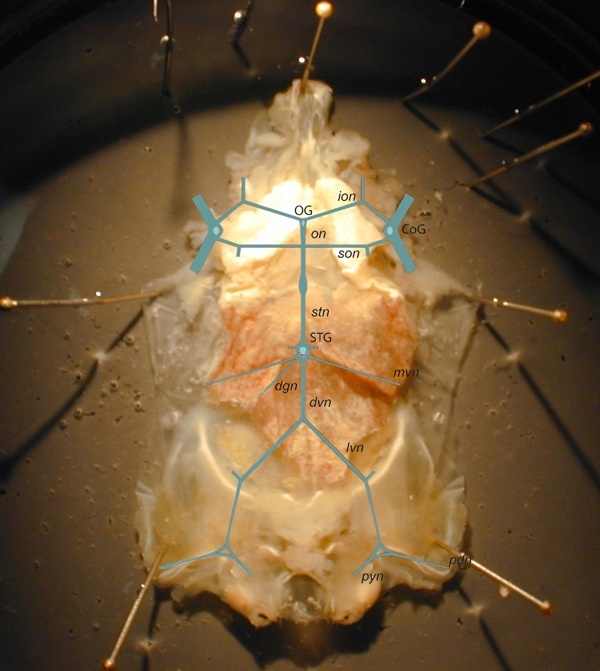


**Figure 1: **Diagram illustrating the approximate placement of the STNS in the stomach prior to dissection.


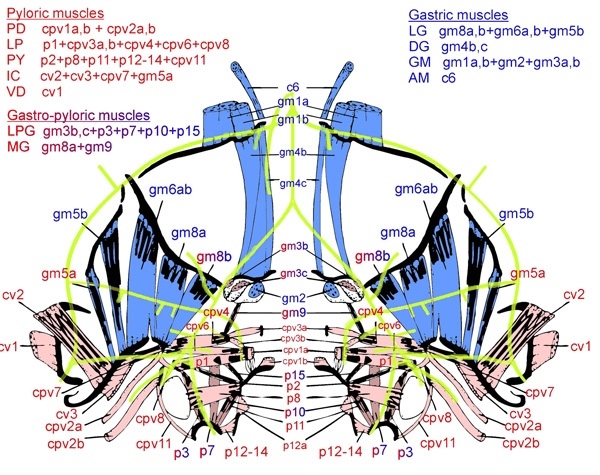


**Figure 2: **Diagram of the various muscles in the lower part of the stomach and an overlay of the STNS in light green. Each cell type in the STG is listed with the muscles they are known to innervate. This map is helpful when planning to dissect a nerve which carries a signal from a given cell (courtesy of the Nadim lab).


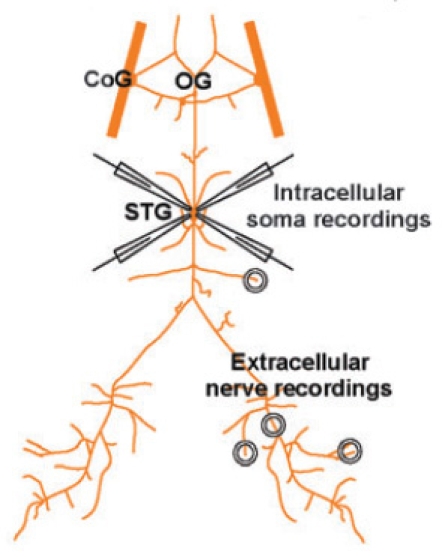
 **Figure 3: **Schematic diagram of the STNS with intracellular electrodes and extracellular wells (from Marder and Bucher, 2007).

## Discussion

The STNS dissection is the first step in performing an electrophysiology or immunohistochemistry experiment, or for obtaining cells for cell culture. Regardless of the intended experiment, the gross dissection will remain unchanged. However, the fine dissection may vary. It is important to plan ahead of time which cells and which nerves you will record from. Most neurons in the STG have processes that innervate at least one muscle in the stomach (see figure 2) and are classified according to their innervated target.  Therefore, extracellular recordings are required to fully verify the identity of a cell that is being recorded intracellularly. The pyloric rhythm’s frequency and robustness are used to monitor the health of the preparation. Therefore, many experiments include extracellular recordings from the *lvn*. The* dgn* is commonly used to monitor the gastric rhythm. The *stn* is often crucial to most experiments because there are many modulatory cells in the anterior end of the STNS and severing that nerve can cause the pyloric and gastric rhythms to cease or become irregular.

Even though the order in which the steps in the fine dissection are done is not important, we find it easier to remove the STNS without damage by keeping the ends of the peripheral nerves anchored to the muscle until after the STG has been disconnected from the surrounding tissue. This keeps the STG and *stn* taut enough to safely and easily remove the muscles and tissue surrounding them.

Once the preparation is in the clear Sylgard dish, any tissue that remains on the STNS should be removed to avoid its interference with the electrodes during an experiment. It is also easier to get good, stable recordings when the preparation is pinned down as taut as possible without causing damage to the nerves. It is particularly important that the STG be pinned down securely by the *alns* with short pins to provide the intracellular electrodes access to as many cells as possible. It is preferable to dissect out at least part of any of the nerves that originate from the STG even if those nerves are not needed for recording. They can provide extra anchors further stabilizing the STG on the dish. It is best to keep them fairly short for maximum support.

## References

[B0] Harris-Warrick RM, Marder E, Selverston AI, Moulins M (1992). Dynamic Biological Networks: The stomatogastric nervous system.

[B1] Marder E, Bucher D (2007). Understanding circuit dynamics using the stomatogastric nervous system of lobsters and crabs. Annu. Rev. Physiol.

[B2] Maynard DM, Dando MR (1974). The structure of the stomatogastric neuromuscular system in Callinectes sapidus, Homarus Americanus and Panulirus argus (Decapoda crustacean). Philos. Trans. R. Soc. Lond. B Biol. Sci.

[B3] Selverston AI, Russell DF, Miller JP (1976). The Stomatogastric Nervous System: Structure and function of a small neural network. Prog. Neurobiology.

